# Spontaneous oscillation in cell adhesion and stiffness measured using atomic force microscopy

**DOI:** 10.1038/s41598-018-21253-9

**Published:** 2018-02-13

**Authors:** Hanna Sanyour, Josh Childs, Gerald A. Meininger, Zhongkui Hong

**Affiliations:** 1 Department of Biomedical Engineering, University of South Dakota, Sioux Falls, SD USA; 2 BioSNTR, Sioux Falls, SD USA; 3 Dalton Cardiovascular Research Center, Department of Medical Pharmacology and Physiology, University of Missouri, Columbia, MO USA

## Abstract

Atomic force microscopy (AFM) is an attractive technique for studying biomechanical and morphological changes in live cells. Using real-time AFM monitoring of cellular mechanical properties, spontaneous oscillations in cell stiffness and cell adhesion to the extracellular matrix (ECM) have been found. However, the lack of automated analytical approaches to systematically extract oscillatory signals, and noise filtering from a large set of AFM data, is a significant obstacle when quantifying and interpreting the dynamic characteristics of live cells. Here we demonstrate a method that extends the usage of AFM to quantitatively investigate live cell dynamics. Approaches such as singular spectrum analysis (SSA), and fast Fourier transform (FFT) were introduced to analyze a real-time recording of cell stiffness and the unbinding force between the ECM protein-decorated AFM probe and vascular smooth muscle cells (VSMCs). The time series cell adhesion and stiffness data were first filtered with SSA and the principal oscillatory components were isolated from the noise floor with the computed eigenvalue from the lagged-covariance matrix. Following the SSA, the oscillatory parameters were detected by FFT from the noise-reduced time series data sets and the sinusoidal oscillatory components were constructed with the parameters obtained by FFT.

## Introduction

Atomic force microscopy (AFM) is a powerful technique with a broad range of biological applications in morphological and mechanical characterization at the molecular^1^, cellular^2–7^, and tissue level^8–10^. Application of AFM in real-time monitoring of cellular mechanics and morphology may provide us with a direct insight into the dynamical changes in mechanics and structures of a live cell at nanoscale^2^. Spontaneous oscillations in cell stiffness, adhesion to extracellular matrix (ECM), and the architecture of cytoskeleton have been reported for multiple cell types^2,11–14^. Preliminary studies have revealed that vasoactive agonists may induce the changes in oscillation of cell elasticity and adhesion to the ECM, however, the exact mechanisms and the biological functions of these oscillations in cell stiffness and adhesion remain to be elucidated^2,15^. Evidence suggests that the mechanism for spontaneous oscillations in cell stiffness and adhesion may be attributed to myosin motors and myosin light chain kinase (MLCK) activity^11,13^. The study of periodic oscillations in live cells is still in its infancy, and thus the physiological, functional, and clinical relevance of these oscillations remain unknown. It is conceivable that the AFM is ideally suited to investigate this temporal aspect of live cell mechanics. However, the highly dynamic characteristics of the oscillations in cell mechanics and high diversity of individual samples necessitate unique analytical approaches for large sets of AFM data. The summarized oscillatory parameters obtained from the analysis of such data sets ultimately reveal the nature of spontaneous oscillations in cell mechanical and adhesive behaviour. Based on the size and complexity of these data sets, an automated analytical approach is required to systematically extract periodic signals from a large set of time series AFM data. This will provide a more unbiased approach to quantitatively investigate and interpret the dynamic live cell mechanics. In previous publications, we applied eigen decomposition that isolated major oscillatory components and reconstructed the time series of AFM data with the filtered oscillation signals^2,13,14^. Here, we describe an approach with the combination of singular spectrum analysis (SSA) and fast Fourier transform (FFT) to investigate the oscillations in cellular mechanics in detail.

SSA was initially introduced by Broomhead and King as a novel technique for the time series data analysis^16,17^. Thereafter, a series of representative publications brought SSA to the center stage as an effective analytical tool applied in dynamical systems and signal processing^18–22^. Over the past decade, SSA has gained growing attention in the analysis of time series data in biological systems such as gene expression^23–25^ and ultrasonic detection and imaging^26^. The method presented here is an automated analytical tool based on the theory of time series data analysis described by Elsner *et al*.^27^ and Golyandina *et al*.^28^. It includes uneven time series cell adhesion and cell stiffness data interpolation, SSA filtering, and FFT prediction. This type of analysis is of fundamental importance for studying these oscillatory events and will help reveal their physiological function and clinical relevance.

## Results

### Detrending and standardization of the time series cell adhesion force

**Figure 1:**
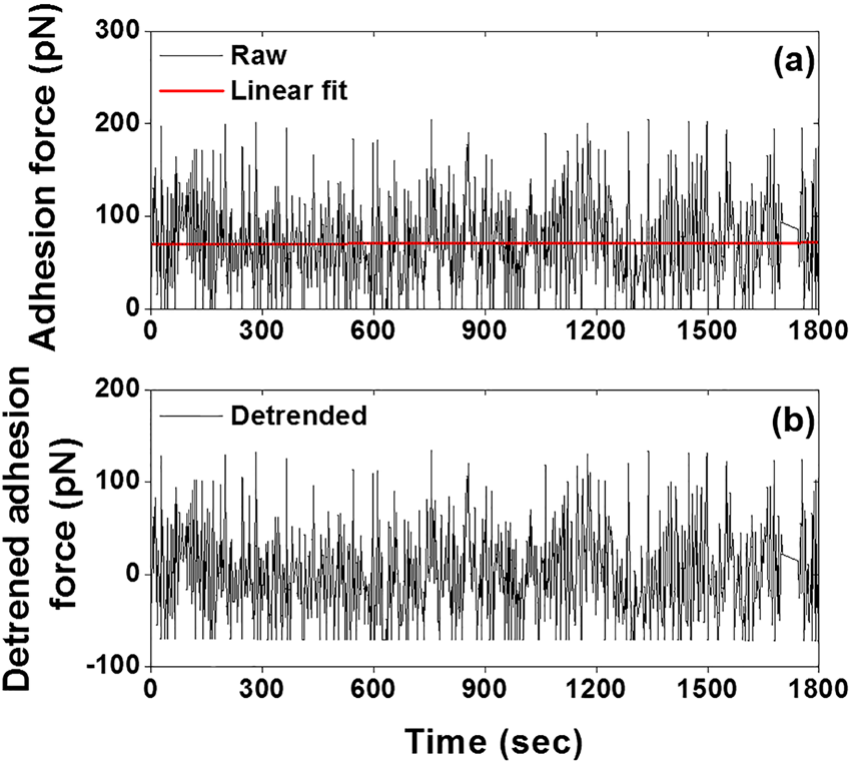
Detrending and normalization of the raw time series integrin-FN total adhesion force per curve. (**a**) Time series of total adhesion force recorded at 0.5 Hz sampling frequency for 1800 sec (n = 1). The red line represents a linear fit of the experimental data set. The trend and intercept of the standardized adhesion time series were computed from the linear fit. (**b**) The detrended and standardized time series, which was used to perform singular spectrum analysis (SSA).

A representative raw time series cell adhesion force recorded with AFM at 0.5 Hz indentation frequency for 1800 sec was plotted in Fig. 1a. The raw time series data was linear-fitted as shown in panel (a) (red line). Prior to singular spectrum analysis (SSA), the raw time series data set was detrended by subtracting the linear fit, and standardized by subtracting the mean value of the data set and dividing by the standard deviation of the data set (Fig. 1b).

### Singular spectrum analysis, grouping of components, and diagonal averaging

**Figure 2:**
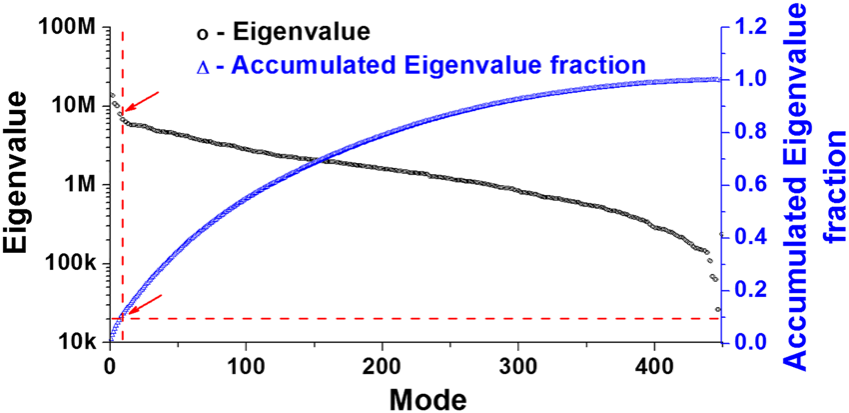
Representative singular spectrum of the standardized time series total integrin-FN adhesion force per curve. The eigenvalues (black circle) were plotted in descending order. The embedding dimension of this individual time series data is 451. The half-length of time series data is normally taken for the dimension of the embedded window. The accumulated eigenvalues (blue triangle) will be used to find the leading components from the standardized time series data. The red arrows indicate the eigenvalue threshold between the biological signal and noise floor.

**Figure 3:**
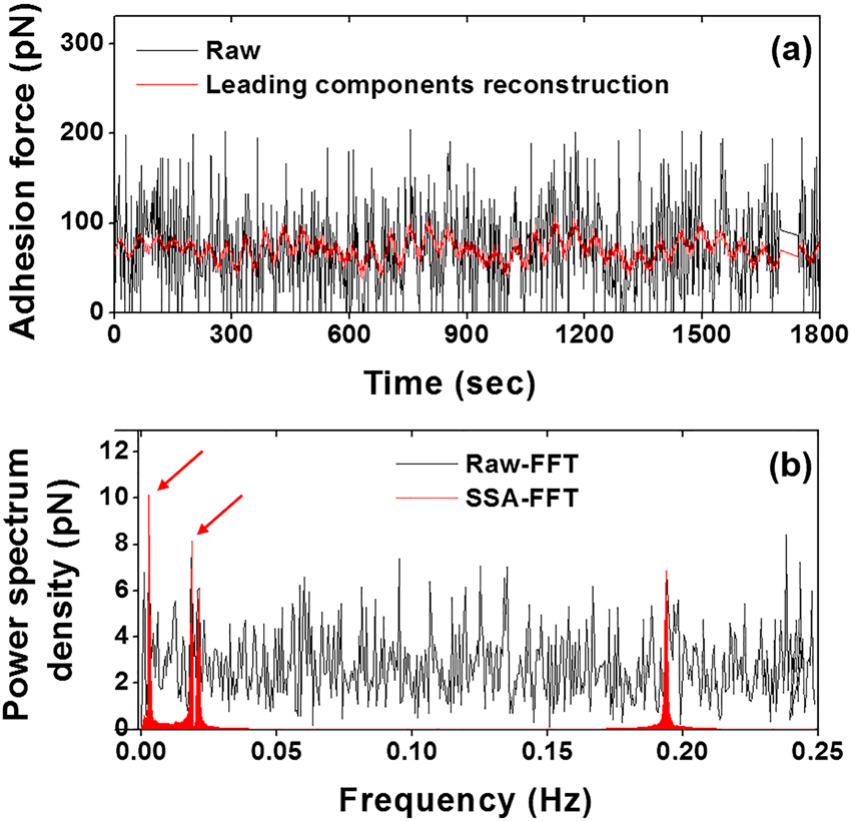
Integrin-FN adhesion force time series reconstruction and its fast Fourier transform (FFT). (**a**) The raw total adhesion force and the SSA filtered leading components of the time series (red line) corresponding to the leading eigenvalues in Fig. 2. The leading components were constructed using eq. 5. (**b**) A representative FFT conducted on the raw data and SSA filtered time series data sets, respectively. The FFT results demonstrated a strong background noise for the raw data compared to the clean background for SSA filtered signal (red). The FFT of the eigen decomposition revealed the frequency and amplitude of the leading oscillatory components with the largest power. In this study, the first two leading components were considered the real biological signals for further analysis (indicated by red arrows).

**Figure 4:**
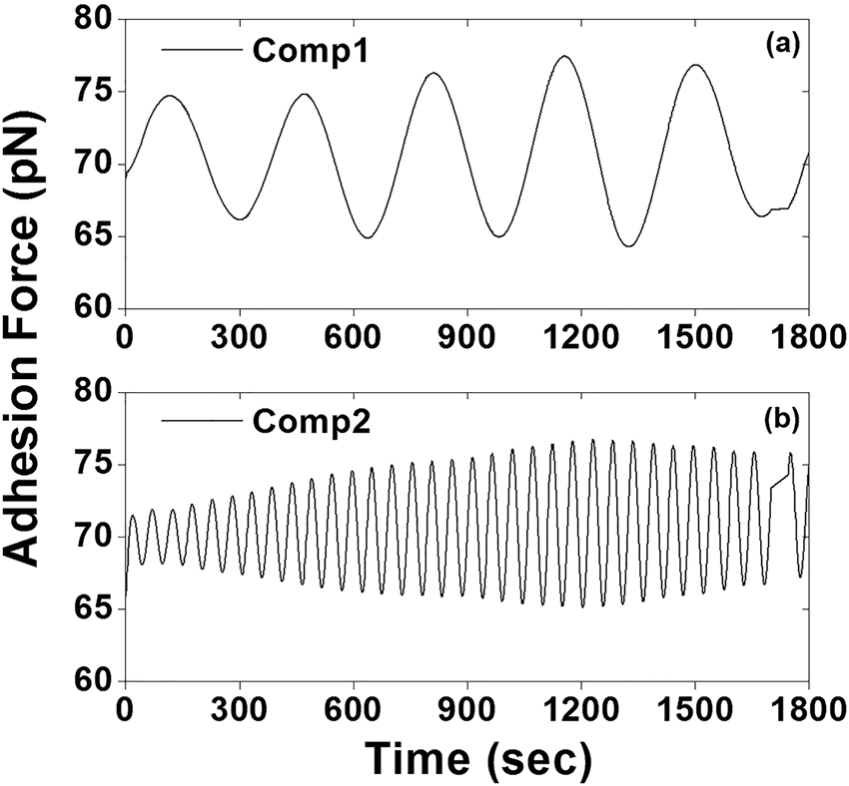
The two principle oscillatory components isolated from the raw time series integrin-FN adhesion force using SSA, which correspond to the two-leading frequencies with the highest power in the Fig. 3(b). Time series adhesion data was reconstructed with the leading two eigentriple matrices using eq. 5. (**a**) Component 1 and (**b**) component 2 gradually increased oscillation powers and reached the highest oscillation amplitude followed by a gradual decrease in oscillation power.

Prior to periodicity detection, singular value decomposition was performed to filter the thermal and mechanical noise and isolate the real biological signals from the time series cell adhesion data. Construction of trajectory matrix of the data series is the first step in SSA methodology. In this study, the trajectory matrixes were constructed with the forward-backward method (eq. 1). The eigenvalues were plotted in the descending order in the singular spectrum of the time series cell adhesion force (Fig. 2, black circle). In addition, the eigenvalues were accumulated until the threshold was achieved (Fig. 2, blue triangle). The red arrows indicate the threshold (0.1 for total adhesion force/per curve) between the potential biological signal and noise floor, i.e., the leading eigenvalues before the threshold (including threshold) were considered real biological signals, whereas eigenvalues beyond the threshold were considered White noise. The SSA-filtered time series adhesion force (Fig. 3a, red solid line) was reconstructed by diagonal averaging the principal eigentriple matrixes *Y*_*I*_ using eq. 5, corresponding to the leading eigenvalues of the trajectory matrix. The FFT power spectrum density (PSD) for raw total adhesion force was compared to SSA filtered time series data set (Fig. 3b). The results showed most of the background noise had been removed after SSA filtering, and it was easy to detect the main leading oscillatory components of the data set, which were the potential candidates for the real biological signals. The oscillation component at 0.194 Hz was too fast for a live cell biomechanics and the components at 0.0186 Hz and 0.0208 Hz were too close to each other. Therefore, in this individual cell, the leading components at 0.0027 Hz and 0.0186 Hz with the highest oscillation powers, as indicated with arrows, were considered the real biological signals for further signal processing (Fig. 3b). Both two leading oscillatory components reconstructed with the two leading eigentriple matrices using eq. 5 gradually increased the oscillation power, reached their highest oscillation amplitudes, and then followed by a gradual decrease in oscillation power (Fig. 4).

### Periodicity detection by fast Fourier transform

**Figure 5:**
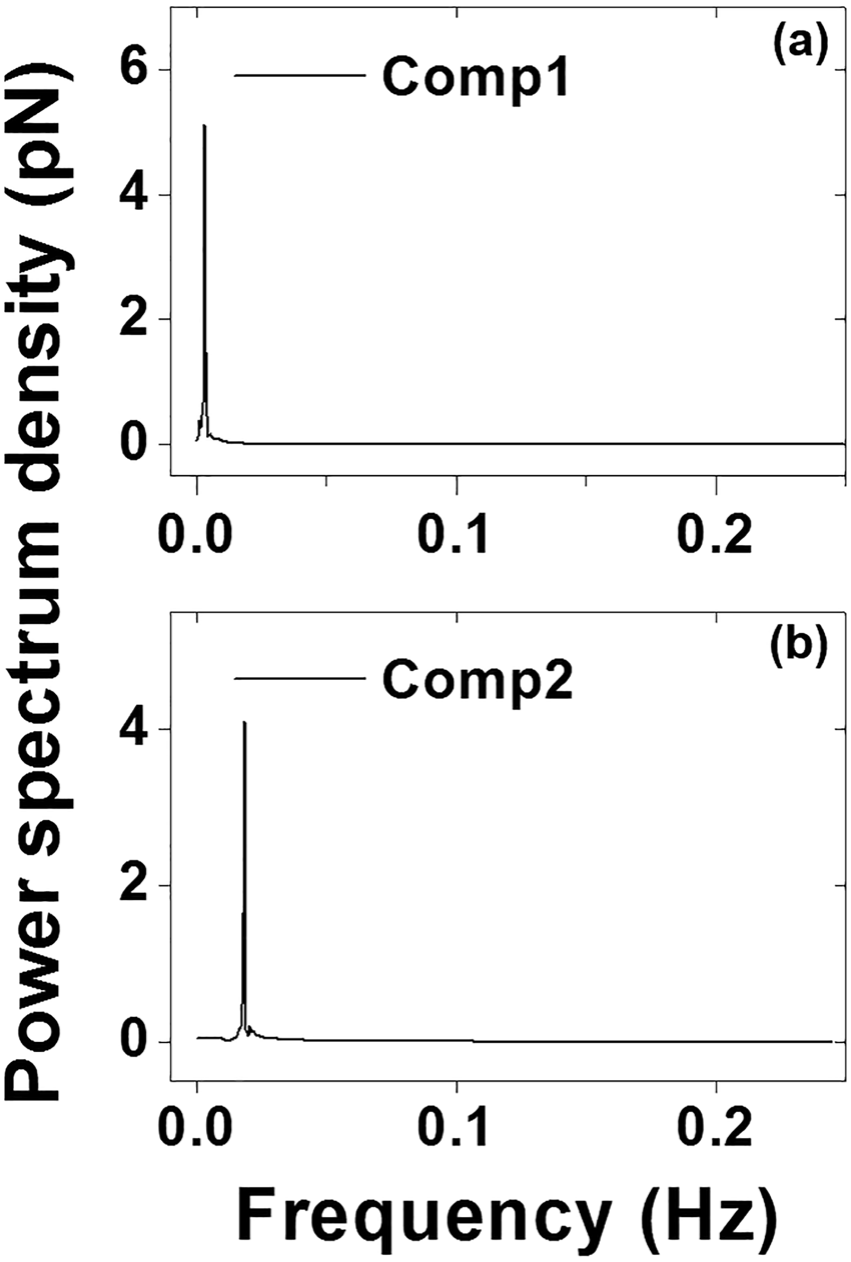
Power spectrum densities plotted using the results of FFT conducted on oscillatory components 1 and 2 in Fig. 4, respectively. Both (**a**) component 1 (0.0027 Hz) and (**b**) component 2 (0.0186 Hz) showed a sharp peak without any side peak or shoulder around them. It indicated that neither component 1 nor component 2 mixed with any other oscillatory signal and that components 1 and 2 could be clearly isolated from background noise.

**Figure 6:**
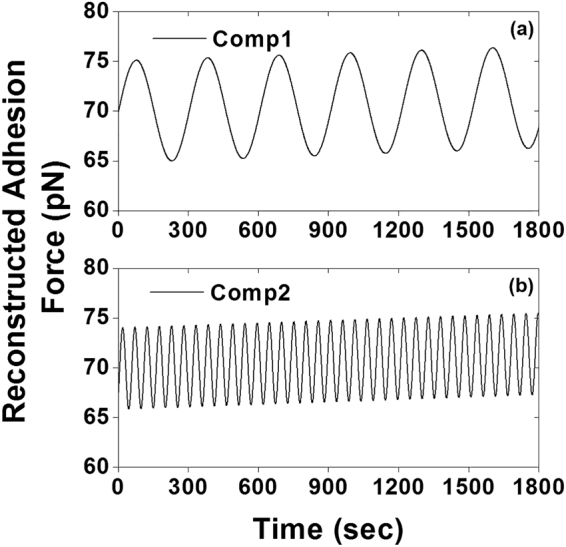
Sinusoidal time series integrin-FN adhesion force was reconstructed for the leading components using the oscillation parameter obtained from the FFT results. Both (**a**) component 1 and (**b**) component 2 oscillate with the average value around 70 kPa.

Periodic signals in the leading two components were detected using FFT and the power spectrum densities were plotted in Fig. 5. Both component 1 and component 2 exhibited sharp peaks at 0.0027 Hz and 0.0186 Hz, respectively, and no side peak or shoulder appeared around them (Fig. 5). This indicates that component 1 and component 2 can be clearly isolated from the noise background. Sinusoidal time series adhesion force for the leading components 1 and 2 were reconstructed using the oscillatory parameter (frequency and amplitude) obtained from the FFT results (Fig. 6).

### Comparison of the oscillations in cell adhesion between live and fixed VSMCs

**Figure 7:**
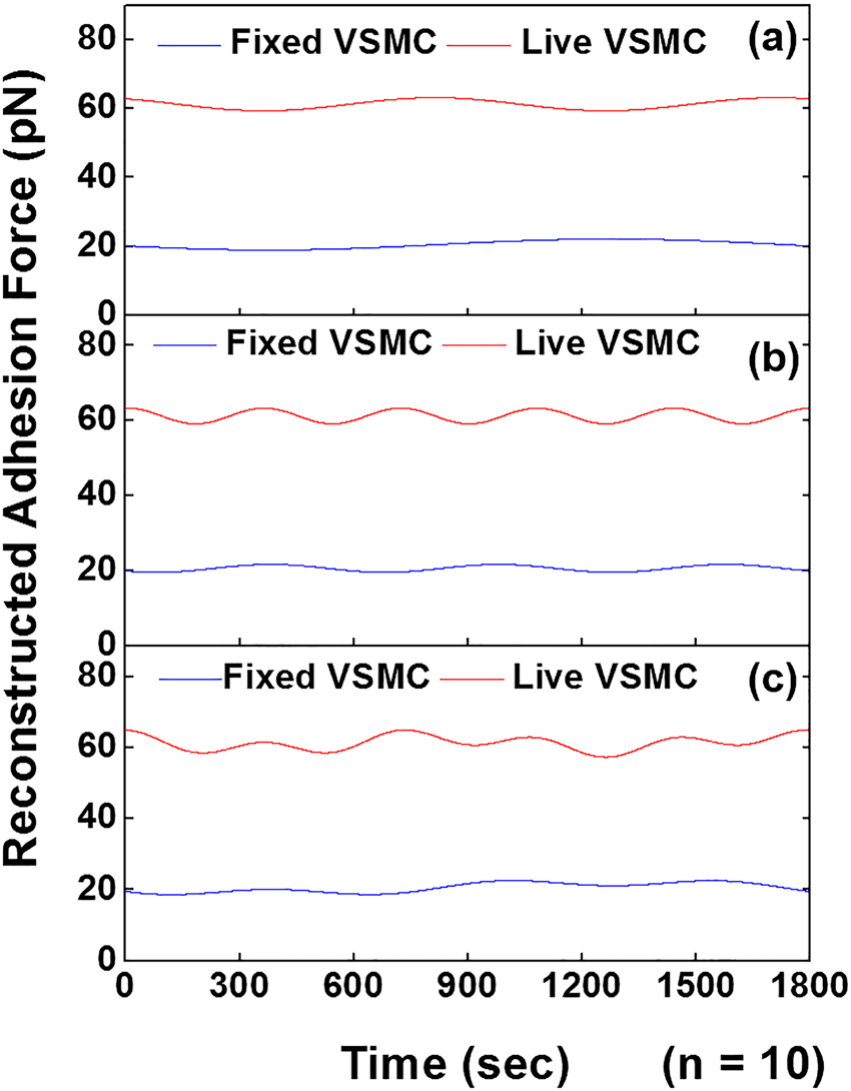
Sinusoidal reconstruction of the time series integrin-FN total adhesion force. (**a**) Oscillatory component 1, (**b**) component 2 and (**c**) the sum of the two leading components for live VSMCs (red) and fixed VSMCs (blue). Time series adhesion data were plotted using the oscillation parameters for the first two leading components obtained from the summarized result of the SSA-FFT performed on the two group samples, respectively (n = 10). The result demonstrated the significant oscillations in the time series adhesion force for live VSMCs compared to the relative flat profile of the total adhesion force for fixed VSMCs.

To verify the leading oscillatory components are the real biological signals generated by a live cell, a sham control experiment was conducted on fixed VSMCs (Fig. 7). Oscillatory components for live VSMCs (red) and fixed VSMCs (blue) were plotted using the oscillation parameters for the first two leading components obtained from the summarized results of the SSA-FFT for the two group samples (Fig. 7a and b), respectively. The results showed distinct oscillations in the time series adhesion data for live VSMCs compared to the relative flat oscillation pattern shown in the real-time adhesion force for fixed VSMCs. The summation of the first two leading oscillatory components for the live cells also exhibited a strong oscillation in cell adhesion force compared to the fixed cells (Fig. 7c). In addition, it can be noted that the reconstructed time series adhesion for live VSMCs showed an average total adhesion force above 60 pN for components 1 and 2. However, the reconstructed time series adhesion data for fixed VSMCs presented a relative low average adhesion force around 20 pN. These results suggest that the oscillations observed in the cell adhesion were generated by the real biological activity of live VSMCs.

### Comparison of the oscillations in live cell E-Modulus  to that of polyacrylamide gel substrate

**Figure 8:**
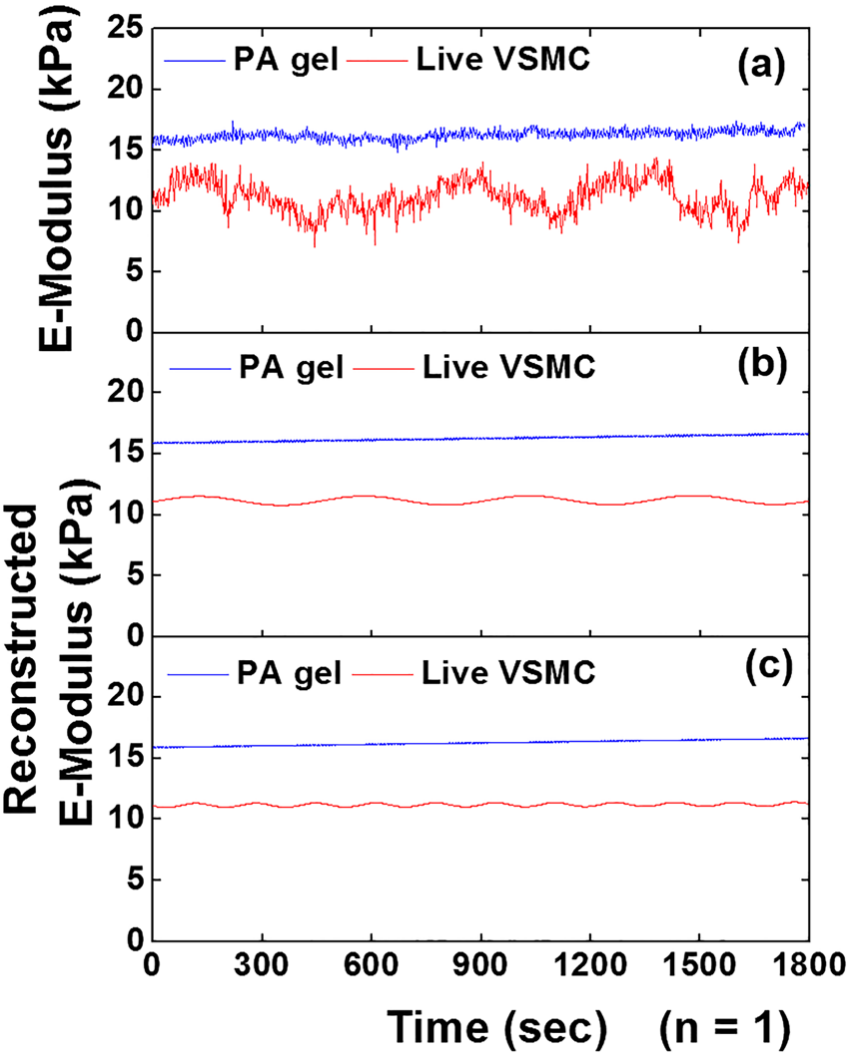
Comparison of the oscillations in E-modulus between a live VSMC and polyacrylamide (PA) gel. (**a**) Representative raw time series of E-modulus data sets of a live VSMC (red) and a PA gel (blue). (**b**) The sinusoidal leading component 1 and (**c**) component 2 of the time series E-modulus reconstructed with SSA-FFT for a live VSMC and PA gel, respectively. The live VSMC showed notable oscillations in the time series E-modulus whereas the control PA gel presented a constant E-modulus during measurement.

**Figure 9:**
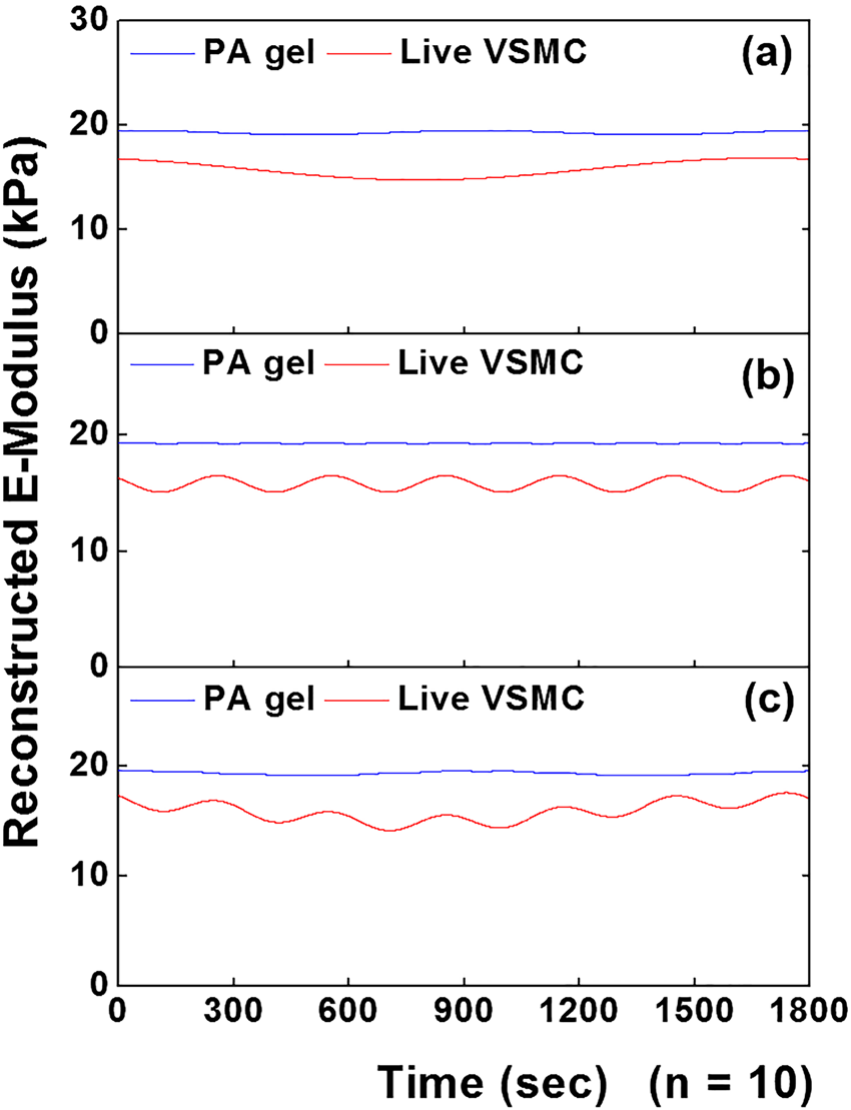
Sinusoidal reconstruction of E-modulus time series. (**a**) Oscillatory component 1, (**b**) component 2, and (**c**) sum of the two leading components for live VSMCs (red) and PA gel control (blue) (n = 10). The sum of components were plotted using the oscillation parameters obtained from the summarized result of the SSA-FFT process, respectively. The result showed the notable oscillations in the time series E-modulus for live VSMCs compared to the constant E-modulus for PA gel during the measurement.

The oscillation behavior of a live VSMC E-modulus was compared to that of a PA gel substrate (Fig. 8). The live VSMC showed a notable oscillation around 10 kPa, whereas the control PA gel exhibited a constant E-modulus around 16 kPa during the entire course of the experiment (Fig. 8a). The time series of the E-modulus of a live VSMC was subjected to the SSA and FFT and two major oscillatory components were isolated and reconstructed similar to the total adhesion force (Fig. 8b and c). To compare the oscillation behaviors in cell E-modulus with the total adhesion force of VSMCs, the group average of the real-time E-modulus of VSMCs (n = 10) was summarized (Fig. 9). The two major oscillatory components of the grouped VSMCs E-modulus showed notable oscillations around 16 kPa, whereas, the PA gel elasticity gave a constant value of 18kPa.

## Discussion

Periodic oscillations in cell adhesion and stiffness have been reported previously by our laboratory^2,13,14^ and others^11,12^. These highly dynamic characteristics of live cells are believed to be associated with the biological functions within the cell. We developed an analytical method by which the temporal aspects of time series cell adhesion force and cell stiffness can be analyzed to extract individual oscillatory components^2,13,14^. In the present study, we utilized FFT to detect oscillations from cell adhesion and cell stiffness data series recorded by AFM. The strength of this approach is that it permits the isolation of oscillatory components for a deeper mechanistic and functional study. The most critical step in this method is SSA filtering to reduce environmental noise embedded within the AFM data series. This allows the periodicity-detecting tool, FFT, to identify the periodic biological signals, and estimate the oscillatory parameters from live cells. SSA filtering is accomplished by choosing a small number of leading eigenvalues and their associated eigenvectors. Following the principle of SSA, small eigenvalues were normalized to zero. A central part of SSA is the construction of a trajectory matrix. In this study, we composed the forward-backward trajectory matrixes with eq. 1. The forward-backward trajectory matrix is typically used to linearly predict short length data set instead of the forward or backward matrix simply because it can increase the number of equations and consequently improve the accuracy for estimated autoregressive coefficients^24,29^. Our results demonstrated that the forward-backward trajectory matrix was able to isolate clear and smooth principal components for periodicity detection through FFT (Fig. 6). Initially, the real biological components entangled with a huge number of periodic thermal and mechanical signals and thus were undistinguishable from these background noises (Fig. 3b). However, the reconstructed total adhesion time series of the leading components resulted in a very sharp and almost noise-free power spectrum density. To test our hypothesis that these oscillations in biomechanics correlate with biological functions of a live cell, we compared the oscillation pattern of cell adhesions between live and fixed VSMCs. Obtained oscillatory signals from live VSMCs showed a distinctly higher frequency and amplitude than the fixed VSMCs, which confirmed the biological origin of these oscillatory signals (Fig. 7). In addition to the difference in oscillation patterns between live and fixed VSMCs, the live VSMCs demonstrated a significantly higher total adhesion force than fixed cell, indicating a loss of integrin-ligand binding capacity in fixed cell.

To verify the oscillations in cell stiffness did not result from the background noise of the AFM system either, the SSA-FFT was also performed on the real-time E-modulus of live VSMCs and PA gel substrate (Fig. 8). The results showed the significant differences in oscillation frequencies and E-Modulus between live VSMCs and PA gels. The grouped summary of the comparison between the live VSMCs and PA substrate, further confirmed that the oscillations in cell E-modulus correlated with the biological signal from a live cell (Fig. 9). Interestingly, the leading components 1 and 2 of the time series of cell adhesion and E-modulus oscillate at similar frequencies correspondingly. It may indicate the underlying correlation between the oscillation in cell adhesion and cell E-modulus. Taken together, these results demonstrate that the combination of SSA and FFT can be used to detect the periodic biological signals from the time series of cell adhesion and E-modulus data sets collected by AFM.

In summary, adhesion force data recorded by AFM carries various environmental noises such as mechanical and thermal instabilities entangled with potential intrinsic biological oscillatory signals due to the noise sensitivity of an AFM device. Utilization of SSA in the real-time AFM data set enables us to reduce the noise level by isolating the principal oscillatory signals from the raw time series adhesion data and reconstruct the time series adhesion force and E-modulus only with the principle oscillatory components. FFT could be applied to detect the periodicity in the SSA pre-filtered noise-free/noise-reduced cell adhesion data set. The method developed in this study allows us to analyze the temporal aspect of real-time cell adhesion and E-Modulus data component-wise, to understand what drives the oscillations, and to investigate the biological functions and clinical relevance of these biomechanical oscillations in live cells.

## Methods

### VSMCs isolation and culture

Sprague-Dawley rats were used for this study and maintained in accordance with the 8th Edition of the Guide for the Care and Use of Laboratory Animals (NRC, 2011). Animals used in these studies were approved by the Laboratory Animal Use Committee of the University of South Dakota (protocol #: 13-09-15-18 C). VSMCs were enzymatically isolated from the descending thoracic aorta of euthanized rats using CO_2_ asphyxiation^2,30^ and seeded onto 60 mm tissue culture dishes (World Precision Instruments, Sarasota, FL). Cells were maintained in DMEM/F-12 (Invitrogen) supplemented with 10% FBS (Atlanta Biologicals, Lawrenceville, GA), 10 mM HEPES (Sigma, St. Louis, MO), 2 mM L-glutamine, 1 mM sodium pyruvate, 100 U/ml penicillin, and 100 μg/ml streptomycin in a humidified incubator (Heraeus Instruments, Newtown, CT) with 5% CO_2_ at 37 °C. The cells used in AFM experiments were maintained in primary culture for 3–7 days without passage. All reagents were purchased from Invitrogen (Life Technologies Carlsbad, CA) except HEPES and FBS.

### Fibronectin coating on AFM probes for Integrin α5β1 mediated cell adhesion measurement

A stylus AFM probe (MLCT, Santa Barbara, CA; Bruker Corp.) was coated with fibronectin (FN, Invitrogen, Carlsbad, CA) using the protocol described in previous publications^2,30^. Briefly, the probe was incubated with 10 mM polyethylene glycol (PEG, Sigma, St. Louis, MO) for 5 min, washed with phosphate buffered saline (PBS), and then incubated with FN (0.1 mg/ml) for 5 min followed by rinsing with PBS.

### Real-time α5β1 integrin-mediated cell adhesion and stiffness measurement using AFM

Real-time monitoring of biomechanical properties of live VSMC was performed using an Asylum AFM System (Model MFP-3D-BIO, Asylum Research, Santa Barbara, CA) mounted on an inverted microscope (Model IX81, Olympus America Inc.). A 5 μm diameter glass microbead was glued to an AFM probe (MLCT-O10, Santa Barbara, CA; Bruker Corp.) and used for E-modulus measurement. All AFM measurements were conducted at room temperature (~25 °C) in CO_2_ independent medium (Invitrogen) without antibiotics. The parameters employed were a 0.5 Hz sampling frequency, with an approach/retraction velocity of 1 µm/sec, 1000 nm traveling distance for one sampling cycle (indentation and retraction), and approximately 1000–3000 pN loading force resulting in an average cellular indentation of 200 nm. Cells were randomly selected and indented at a site between the nucleus and cell boundary to collect approximately 900 force curves within 30 min. To minimize drifting, after the probe was submerged in cell bath, the AFM system was thermally and mechanically equilibrated for at least 30 min. Each cantilever was calibrated after a given adhesion and before a stiffness experiment using thermal noise amplitude analysis^31,32^.

### AFM force curve analysis

AFM force curve analysis was automated using a proprietary software package written in MATLAB (R2016a, Mathworks). Adhesion forces between FN and integrin adhesion complexes were determined by the product of cantilever spring constant and the height of ruptures in retraction force curve. The cell stiffness was estimated by fitting a modified Hertz model to the approaching force curve as described in our previous publication^2,30^. From retraction force curves, a number of cell adhesion properties can be evaluated such as adhesion probability, average adhesion force per curve, total adhesion force per curve and loading rate of cell adhesion force (Fig. S1).

### Singular value decomposition of the time series AFM data

The first step in the SSA algorithm is embedding the initial time series adhesion data with a window matrix, and converting it into a trajectory matrix. Given the time series of adhesion data with the elements *x*_1_, *x*_2_, *x*_3_, …, *x*_*N*_, the forward-backward trajectory matrix *T* was composed by eq. 1. The forward-backward trajectory matrix is normally used to estimate the coefficient of autoregressive (AR) linear prediction, where each sample of a time series data is approximated with a linear combination of previous samples and AR coefficients. This method is able to double the number of equations for AR coefficient estimation and thus increase the accuracy of the output coefficient for short data series^29,33–35^.

1 $$T=[\begin{array}{llll}{x}_{p} & {x}_{p-1} & \ldots  & {x}_{1}\\ {x}_{p+1} & {x}_{p} & \ldots  & {x}_{2}\\ \,\vdots  & \,\vdots  & \ddots  & \,\vdots \\ {x}_{n-1} & {x}_{n-2} & \ldots  & {x}_{n-p}\\ {x}_{2} & {x}_{3} & \ldots  & {x}_{p+1}\\ {x}_{3} & {x}_{4} & \ldots  & {x}_{p+2}\\ \vdots \, & \vdots \, & \ddots  & \vdots \,\\ {x}_{n-p+1} & {x}_{n-p+2} & \ldots  & {x}_{n}\end{array}]$$

where *p* is the order of the AR model, and in this study *p* = *N*/2, *N* is the number of data in the time series.

Singular value decomposition (SVD) of the trajectory matrix is the central part of the SSA, which is performed by the eigenvalue decomposition (EVD) of the covariance matrix. Various approaches to the construction of the covariance matrix have been reported by Broomhead *et al*.^17^ and Vautard *et al*.^20^. In this study, we composed the covariance matrix by the product of the trajectory matrix and its transpose as defined by eq. 2, which was originally proposed by Broomhead *et al*.^17^.

2 $$C=T\cdot T\text{'}$$

where *C* is the covariance matrix, *T* is the trajectory matrix of the original time series adhesion force and *T*′ is the transpose of trajectory matrix *T*.

EVD produced *L* many of eigenvalues of covariance matrix *C*, denoted by $\,{{\boldsymbol{\lambda }}}_{i},(1\le i\le L)$, and left and right eigenvectors, denoted by *U*_*i*_,(1 ≤ *i* ≤ *L*) and *V*_*i*_, (1 ≤ *i* ≤ *L*) respectively. The trajectory matrix *T* was reconstructed by eq. 3.

3 $$T=\sum _{i=1}^{d}{U}_{i}\sqrt{{{\boldsymbol{\lambda }}}_{i}}{V}_{i}^{T}$$

where *d* is the maximal value of *i*, such that ${{\boldsymbol{\lambda }}}_{i} > 0$, the collection ${U}_{i}\sqrt{{{\boldsymbol{\lambda }}}_{i}}{V}_{i}^{T}$ is the *i*^*th*^ eigentriple of the trajectory matrix *T*.

### Reconstruction of time series data by grouping of the leading components

Eigentriples were grouped according to the similarity of the individual components^28^. The grouping step of the SSA consisted of decomposing the *L* × *K* matrix *T* into a number of disjoined subgroups according to the components’ trend and oscillation similarity. In this study, we pair-wisely grouped every two eigentriples for the leading eigentriples corresponding to the leading eigenvalues into principal eigentriple matrixes *Y*_*I*_ (*I* = *1*, *2*, *3*…) using eq. 4 with the dimensions of *L* × *K* for trajectory matrix *T*.

4 $${{\boldsymbol{Y}}}_{{\boldsymbol{I}}}=\sum _{i=I}^{I+1}{U}_{i}\sqrt{{{\boldsymbol{\lambda }}}_{i}}{V}_{i}^{T}\,(I=1,2,3)$$

For the time series cell stiffness data, 0.6 was empirically set as the threshold of the accumulated eigenvalue fraction, namely the eigenvalue prior to this threshold were considered as real biological signals and they contribute 60% of the energy of the oscillation in cell stiffness, and the rest were considered as the background noise. For the time series adhesion data, the threshold was set as 0.1 due to the relatively high background noise generated by the system during cell adhesion measurement compared to the stiffness data (indicated by the red arrow on the blue lin2 in Fig. 2).

Diagonal averaging is the last step of SSA to transform each matrix of the grouped decomposition *Y*_*I*_ into a new time series data with length *N*. The diagonal averaging steps were performed by eq. 5^28^.

5 $${g}_{{I}_{k}}=\{\begin{array}{ll}{Y}_{{I}_{k,P}}, & (k=1)\\ \frac{1}{2k-1}(\sum _{m=1}^{k}{Y}_{{I}_{m,P-k+m}}+\sum _{n=1}^{k-1}{Y}_{{I}_{L+n,k-n}}), & (2\le k\le L-1)\\ \frac{1}{2L-1}(\sum _{m=1}^{L}{Y}_{{I}_{m,P-k+m}}+\sum _{n=1}^{L-1}{Y}_{{I}_{L+n,k-n}}), & (k=L)\\ \frac{1}{2L}(\sum _{m=1}^{L}{Y}_{{I}_{m,P-k+m}}+\sum _{n=1}^{L}{Y}_{{I}_{L+n,k-n}}), & (L+1\le k\le P)\\ \frac{1}{N+L-k}(\sum _{m=2}^{L+1}{Y}_{{I}_{m,m-1}}+\sum _{n=1}^{L}{Y}_{{I}_{L+{\rm{n}},k-n}}), & (k=P+1)\\ \frac{1}{2(N-k)+1}(\sum _{m=k-P+1}^{L}{Y}_{{I}_{m,P-k+m}}+\sum _{n=k-P}^{L}{Y}_{{I}_{L+n,k-n}}), & (P+2\le k\le N-1)\\ {Y}_{{I}_{2L,P}}, & (k=N)\end{array}$$

where *g*_*I*_ is the reconstructed time series component, *Y*_*I*_ is the Grouped Eigentriple matrix constructed by the forward-backward trajectory matrix.

### Periodicity detection by a fast Fourier transform

A fast Fourier transform (FFT) was employed to detect the oscillatory signal of the leading components. The algorithm used for FFT in this study is eq. 6, originally proposed by Cooley and Turkey^36^.

6 $${X}_{k}=\sum _{n=0}^{N-1}{x}_{n}{e}^{-i2\pi k\frac{n}{N}}\,k=0,\ldots ,N-1.$$

where *X*_*k*_ is a series of complex numbers, *x*_*n*_ is the time series data, and the frequency corresponding to the biggest complex modulus (|*X*_*k*_|) was considered as the frequency of the oscillatory component.

### Data availability

The raw data used to generate the figures in this manuscript are available from the corresponding or the first author upon request.

## Electronic supplementary material

Supplementary information
