# Supplementary material for: Spontaneous oscillation in cell adhesion and stiffness measured using atomic force microscopy
Source: Sci Rep. 2018 Feb 13;8:2899. doi: 10.1038/s41598-018-21253-9 (PMC5811453; doi:10.1038/s41598-018-21253-9)
Supplement: Supplementary file 1 — Supplementary information [file 41598_2018_21253_MOESM1_ESM.pdf]

Spontaneous oscillation in cell adhesion and stiffness measured using atomic force microscopy

Hanna Sanyour[1,2*], Josh Childs[1,2*], Gerald A Meininger[3†], Zhongkui Hong[1,2†]

[1]Department of Biomedical Engineering, University of South Dakota, Sioux Falls, SD

[2]BioSNTR, Sioux Falls, SD

[3]Dalton Cardiovascular Research Center, Department of Medical Pharmacology and Physiology, University of Missouri, Columbia, MO

[*]These two authors contributed equally

[†]To whom correspondence should be addressed:

Zhongkui Hong, Ph.D.,

Biomedical Engineering Department,

University of South Dakota,

4800 N Career Ave, Suite 221, Sioux Falls, SD 57107

Fax : (605) 782-3280,

Tel : (605) 275-7468,

E-mail : Zhongkui.Hong@usd.edu

Gerald A. Meininger, Ph.D.,

Dalton Cardiovascular Research Center,

University of Missouri,

134 Research Park Dr., Columbia, MO 65211

Fax: 573-884-4232,

Tel: 573-882-9662,

E-mail: Meiningerg@missouri.edu

Supplementary information

Cell adhesion evaluation from AFM force curve

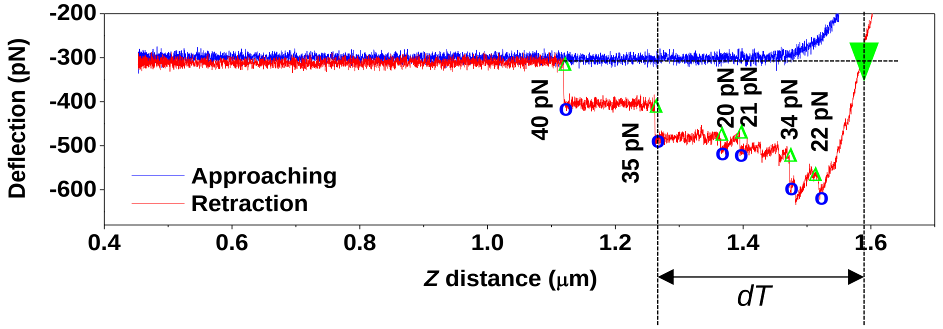

Total adhesion force: 40+35+20+21+34+22 =172 pN
Adhesion probability (Adhesion number per curve): 6
Average adhesion force: 172/6 = 28.7 pN
Rupture length *dT:* the distance between the point where the AFM tip and cell surface establish a contact and the point where the rupture occurred.

Figure S1. Representative AFM force curve showing the approaching curve (blue), and adhesion ruptures and associated forces occurring in the retraction curve (red). The ruptures represented the dissociation between the ECM coated AFM tip and the transmembrane integrins, and were calculated by multiplying the height of the rupture by the cantilever spring constant (Hooke's law). Adhesion probability was defined as the number of the rupture per curve. Total adhesion force was calculated by taking the sum of all detected rupture values. Rupture length *dT* was defined as the distance between the point where the AFM tip and cell surface establish a contact and the point where the rupture occurred.

The real-time distribution of the E-Modulus of a representative live cell around different time points.

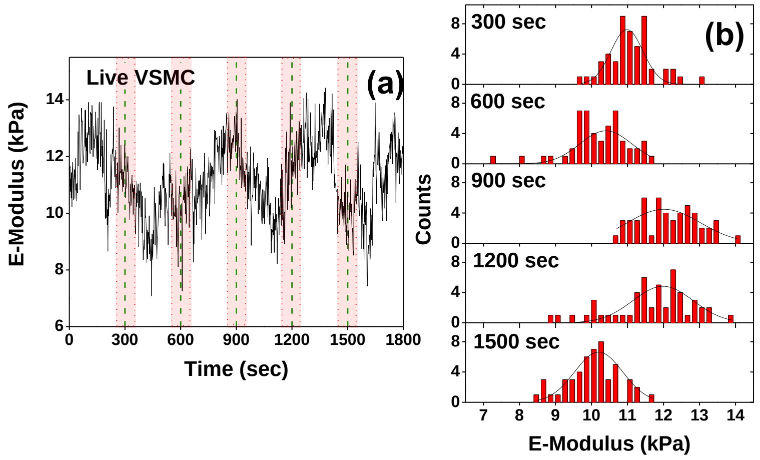

Figure S2. (a) A representative real-time live VSMC E-Modulus for 1800 sec. **(b)** Histograms of a live VSMC E-Modulus distribution at different time points. VSMC E-Modulus distribution histograms were plotted at each time point (in the range of ±50 sec around the time point) as indicated by the pink area in panel (a) and were fitted with a first order Gaussian curve (black curve in panel (b)).

How to average the oscillation components with similar frequency for a group of cells?

Figure S3 displays (panel (a)) an example of the normalized individual real-time E-Modulus, (panel (b)) major oscillation component isolated from corresponding real-time E-Modulus, and (panel (c)) summarization of oscillation components with similar frequency. As shown in the Figure S3(b), the four oscillation components have almost identical oscillation frequency with different amplitude that may linked to the cell stiffness and activity. The oscillation phase of these four components are 2.06, 4.54, 1.37, and 5.26, respectively. The shift in oscillation phase might result from differences the cell cycle or metabolism of each individual cell. For summarizing the individual experiments, we must consider the shift in the oscillation phase of individual cells. If we take the average of these oscillation without the phase adjustment and the

N (number of individual experiment) is big enough, the summarized oscillation amplitude will be diminished since these oscillation components offset each other (blue line in panel (c)). However, if we take the average of these oscillation components after adjusting the oscillation phase of each component, the summarized oscillation amplitude will be the correct average amplitude of these four oscillations amplitude (red line in panel (c)).

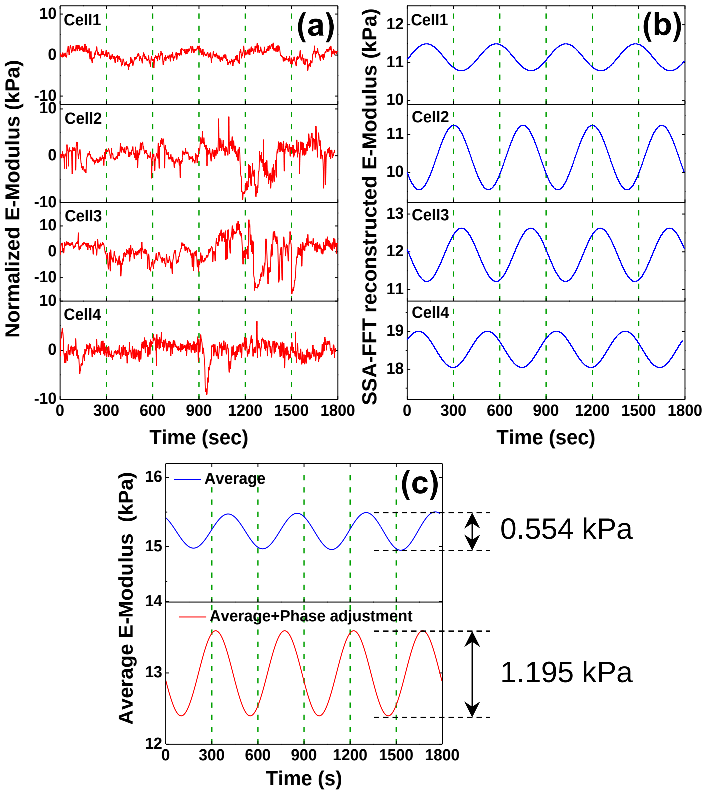

Figure S3. (a) Example of the normalized real-time cell E-Modulus for four individual cells. **(b)** Sinusoidal-reconstructed oscillation component isolated using SSA-FFT from the corresponding experimental data shown in *panel (a)*. The four isolated components from corresponding raw data have similar oscillation frequency but have very difference oscillation phase. **(c)** The blue line is the summarized oscillation by taking average of the four oscillation components without phase adjustment. The red line is the summarized oscillation by taking average of the four oscillation components after phase adjustment.
